# Population dynamics and community structure of *Anopheles* mosquitoes along the China-Myanmar border

**DOI:** 10.1186/s13071-015-1057-1

**Published:** 2015-09-04

**Authors:** Ying Wang, Daibin Zhong, Liwang Cui, Ming-Chieh Lee, Zhaoqing Yang, Guiyun Yan, Guofa Zhou

**Affiliations:** Institute of Tropical Medicine, Third Military Medical University, Chongqing, China; Program in Public Health, University of California at Irvine, Irvine, CA USA; Department of Entomology, Pennsylvania State University, University Park, PA USA; Department of Pathogen Biology and Immunology, Kunming Medical University, Kunming, China

**Keywords:** Malaria vector, China-Myanmar border, Population dynamics, Population density, Species richness, Species diversity, Seasonality

## Abstract

**Background:**

Understanding the ecology of malaria vectors such as species composition and population dynamics is essential for developing cost-effective strategies to control mosquito vector populations.

**Methods:**

Adult mosquitoes (*n* = 79,567) were collected in five villages along the China-Myanmar border from April 2012 to September 2014 using the CDC light trap without bait method. Mosquito community structure, *Anopheles* species composition and diversity were analyzed.

**Results:**

Twenty species of *Anopheles* mosquitoes were identified, with *An. minimus* s.l. accounting for 85 % of the total collections. Mosquito densities varied from 0.05 females per trap per night (f/t/n) to 3.00 f/t/n, with strong seasonality in all sites and densities peaked from June to August. *An. minimus* s.l. was predominant (accounting for 54–91 % of total captures) in four villages, *An. maculatus* s.l. was predominant (71 %) in the high elevation village of Dao Nong, and *An. culicifacies* accounted for 15 % of total captures in the peri-urban area of Simsa Lawk. All 20 species have been captured in the Mung Seng Yang village, 18 and 15 species in Ja Htu Kawng and Na Bang respectively, and nine species in both Simsa Lawk and Dao Nong. Species richness peaked from April to August. Species diversity, species dominance index, and species evenness fluctuated substantially from time to time with no clear seasonality, and varied greatly amongst villages.

**Conclusions:**

Mosquitoes were abundant in the China-Myanmar bordering agricultural area with clear seasonality. Species composition and density were strongly affected by natural environments. The targeted intervention strategy should be developed and implemented so as to achieve cost-effectiveness for malaria control and elimination along the border areas.

**Electronic supplementary material:**

The online version of this article (doi:10.1186/s13071-015-1057-1) contains supplementary material, which is available to authorized users.

## Background

Mosquitoes are vectors of various human and animal infectious diseases [1]. Vector control is an important component and sometimes the only effective way (e.g., for dengue fever) to reduce the transmission of these diseases [1–5]. Understanding the species composition and population dynamics of the local mosquitoes is the crucial step for developing and implementing appropriate strategies to control mosquito vector populations and subsequently the mosquito-borne infectious diseases.

Malaria is one of the major mosquito-borne diseases endemic to East and Southeast Asia [6–8]. The Greater Mekong Subregion (GMS) is one of the most threatening foci of malaria in Southeast Asia [7–10]. The malaria burden in Myanmar is the heaviest among the GMS nations. More than half of the malaria cases and ∼ 75 % of the malaria deaths in the GMS occurred in Myanmar [11]. International border regions, such as the one shared by China and Myanmar’s Kachin State, have the highest malaria incidence and the highest malaria-related mortality rate, at 7.8 deaths/1000 people [11–13]. A large proportion of ethnic minorities live in these remote, often hilly and forested border areas, which are prone to malaria transmission by the forest mosquitoes *Anopheles minimus* s.l. and *Anopheles dirus* s.l.. The Chinese side of the China-Myanmar border remains a significant foci of clinical malaria despite significant decline of malaria in other parts of China in the past two decades [14, 15].

Effective control of disease vectors requires a good understanding of vector ecology, including vector distribution, development and population dynamics. Recent achievements in malaria control and enthusiasm from international communities have inspired a number of East and Southeast Asian countries such as Thailand and China to redirect program strategies from malaria control towards elimination. There is an increased focus on studying malaria transmission along the China-Myanmar border; however, mosquito vector ecology in this area remains almost untouched [16–19]. This lack of information hinders progress toward the goals of eliminating malaria and controlling other mosquito-borne diseases in the area.

The purpose of this study is to acquire critical epidemiological information on vector population ecology by conducting field surveys on vector species composition, species richness, diversity, and population dynamics on both sides of the China-Myanmar border. The results will help to fill important knowledge gaps and aid in developing cost-effective malaria control and elimination strategies in the area.

## Methods

### Study sites

**Fig. 1:**
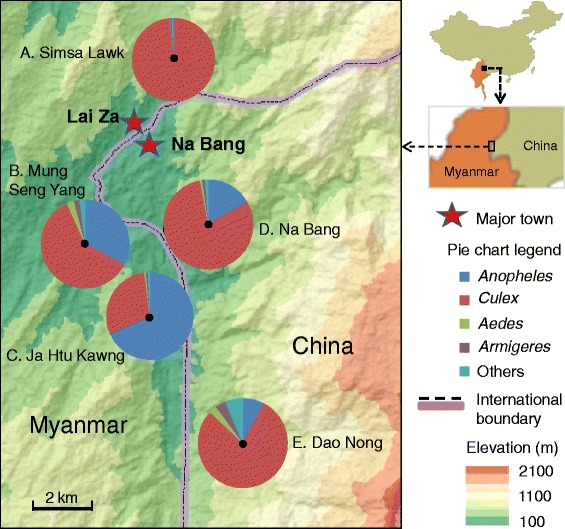
Locations of the study sites and pie-chart showing mosquito community structure (percentage distribution) along China-Myanmar border. Localities: **a** Simsa Lawk; **b** Mung Seng Yang; **c** Ja Htu Kawng, **d** Na Bang; and **e**. Dao Nong

This study was conducted at five sites along the China-Myanmar border (Fig. 1). Two of the sites are located in Yingjiang county, Yunnan province, China: Na Bang village, at an elevation of c. 250 m above sea level (a.s.l.) (range from 240 to 270 m), and Dao Nong village, with an elevation of c. 700 m a.s.l. (range from 660 to 740 m). The other three study sites are in the Lai Za district of Kachin State, Myanmar: Simsa Lawk, representing a peri-urban environment, and Ja Htu Kawng and Mung Seng Yang villages, representing rural settings. All three sites in Myanmar are located in the same valley where Na Bang town and Lai Za town are located, at an elevation of c. 250 m a.s.l. (range from 240 to 280 m). All five study sites are surrounded by mountains and comprise either dense forest (Na Bang and Simsa Lawk), maize cultivations on slopes (Ja Htu Kawng and Mung Seng Yang), or white pepper plantations (Dao Nong village). The major habitat type found in all villages was standing water ponds and fish ponds. The area has a subtropical climate, with January through March being relatively dry and cold, and June to August is considered to be the rainy summer season. The annual rainfall is about 1450 mm with no clear dry season. Annual mean temperature is 21.7 °C, with a low of 15 °C in January and high of 25 °C in July. The caught populations totaled 230, 235, and 510 in 2012 in Mung Seng Yang, Ja Htu Kawng, and Simsa Lawk respectively; the populations were 470 and 240 (census includes permanent residents only) in 2012 in Na Bang and Dao Nong, respectively.

### Mosquito collection and species identification

In the study sites in China, mosquitoes were collected from systematically selected 68 houses in Na Bang village and all 48 houses in Dao Nong village from April 2012 to April 2013. In Myanmar, mosquitoes were collected from systematically selected 31 houses in Simsa Lawk, from 34 houses in Ja Htu Kawng, and from all 40 houses in Mung Seng Yang, from April 2012 to September 2014. Locations of all sampled houses were determined with a handset global positioning system (Additional file 1: Table S1) (Garmin International Inc., Olathe, KS, USA). Owners of the surveyed houses were requested to sign a freely administered informed consent form covering participation in the study, questionnaire surveys, and monitoring of mosquito population dynamics, i.e., permission to set up the traps and collect mosquitoes. To conduct the sampling, CDC miniature light traps (BioQuip Products, Inc, Rancho Dominguez, CA, USA) without bait were placed in houses at dusk and then collected in the following morning. Traps were placed in the same house for two consecutive nights each sampling occasion. A total of 36 traps were deployed each night. Traps shifted from house to house and from village to village over time so that every house in a given village and every village in the study area has been sampled once (2 nights) every two weeks or two times (4 nights) each month. Once collected, mosquitoes were morphologically separated as *Anopheles*, *Culex*, *Aedes*, and other subfamilies or genera.

*Anopheles* mosquitoes were further morphologically identified to species or species complex in the field when possible [20, 21]. Subsample specimen were stored in −20 °C freezer immediately after species or species complex was identified and kept frozen until further analysis. The DNA of *An. minimus* s.l. mosquitoes was extracted from the abdomen of each mosquito for further species confirmation [17]. *Anopheles* collections from Dao Nong, Na Bang, Ja Htu Kawng, and Mung Seng Yang villages between May 2012 to April 2013 have been used in previous publication [17], the previous stud only published the summary of pooled data of all villages and time frames, and it focused on the role of *An. minimus* as the malaria vector [17]. In addition to new collections, species diversity, evenness, similarity and their temporal changes were not determined in the previous study. Inter-village heterogeneity, e.g., differences in *Anopheles* densities, species richness, and diversity, etc. have not been compared.

### Data analysis

Density of mosquitoes was calculated as the number of females per trap per night (f/t/n) and monthly average was calculated over all houses in a given village. Pooled *Anopheles* density was calculated by summing across all *Anopheles* species. Species richness was measured by the number of species. Species diversity of *Anopheles* mosquitoes was measured using the Simpson diversity index, D, which can be interpreted as the probability of interspecific encounter, and Shannon-Weiner index, H, which takes into account of individuals of each species [22–25]. The Simpson diversity index is defined as $D=1-{\displaystyle \sum_{i=1}^N{\displaystyle {p}_i^2}}$, where *p*_i_ is the fraction of a species which belong to the i-th species, and N is the number of species. If all species are equally distributed, then the index has its highest value of [1 − (1/N)]; if one species is dominant, then the index will approach zero. Other indices calculated included the Berger-Parker dominance index, d, evenness index, E = H/Ln(S), where H is the Shannon-Weiner diversity index and S is the total number of species observed at a given village, and the Morista-Hoen similarity index, C [25].

Mosquito abundance data was transformed by the power function $\sqrt[4]{X}$, where *X* is the monthly density, before comparison and this transformation ensured overall data normality as required for data comparison using analysis such as analysis of variance (ANOVA) and Student *t*-test [25, 26]. Differences in monthly average mosquito abundance (time series) among different villages were tested using one-way analysis of variance (ANOVA) with repeated measures after data transformation. Pairwise differences in mosquito abundance were compared using the ANOVA post-hoc Tukey-Kramer HSD test or Hsu’s MCB test with a significance level of 5 %. Statistical analysis was carried out using JMP statistical software (JMP 9.0, SAS Institute Inc., USA).

## Results

Over the study period, a total of 14,786 trap nights were conducted and 79,567 mosquitoes were collected in the five villages. Among the mosquitoes captured, the majority were *Culex* (*n* = 62,828 or 79.0 %), followed by *Anopheles* (*n* = 15,410, 19.4 %), *Aedes* (*n* = 466, 0.6 %); the remainder belonged to other subfamilies or genera (Fig. 1). Twenty *Anopheles* species have been identified (Table 1), *An. minimus* s.l. accounted for 84.6 % of total *Anopheles* collections. All other species accounted less than 5 % of total collections. Thirteen of the 20 *Anopheles* species each accounted for less than 1 % of total *Anopheles* collections (Table 1). PCR results (*n* = 425) confirmed that all *An. minimus* s.l. was *An. minimus* A.

**Table 1:** *Anopheles* species composition by village and pooled across study sites and study period

Species	Composition by village					Pooled	
	DN	NB	JHK	MSY	SSL	N	%
***An. minimus*** **A**	14.73	54.18	90.73	74.02	69.47	13,038	84.61
***An. maculatus*** **s.l.**	71.32	13.80	0.99	4.12	9.47	530	3.44
***An. culicifacies*** **s.l.**	5.04	5.19	2.05	4.56	15.79	437	2.84
***An. jeyporiensis***	2.33	1.01	2.02	1.04	0.53	277	1.80
*An. splendidus*	4.26	5.44	0.87	3.28	0.00	237	1.54
***An. vagus***	0.39	3.16	1.05	2.68	2.11	220	1.43
***An. sinensis***	0.00	3.16	0.61	2.56	0.53	161	1.04
***An. barbirostris*** **s.l.**	0.39	6.58	0.33	1.68	0.00	133	0.86
***An. peditaeniatus***	1.16	2.66	0.25	2.96	0.00	127	0.82
***An. fluviatilis***	0.00	0.00	0.73	0.24	0.00	91	0.59
***An. kochi***	0.00	0.76	0.20	0.36	0.53	39	0.25
***An. tessellatus***	0.00	2.78	0.01	0.64	0.00	39	0.25
*An. crawfordi*	0.00	0.00	0.04	0.24	0.00	11	0.07
***An. lesteri***	0.00	0.00	0.03	0.32	0.00	11	0.07
*An. barbumbrosus*	0.39	0.00	0.01	0.32	0.00	10	0.06
*An. messeae*	0.00	0.63	0.01	0.12	0.00	9	0.06
***An. annularis*** **s.l.**	0.00	0.13	0.02	0.12	0.53	7	0.05
***An. stephensi***	0.00	0.38	0.01	0.04	1.05	7	0.05
*An. indefinitus*	0.00	0.13	0.00	0.08	0.00	3	0.02
*An. gigas*	0.00	0.00	0.00	0.04	0.00	1	0.01
Other *Anopheles* (unidentified)	0.00	0.00	0.06	0.60	0.00	22	0.14

Species of malaria vectors are marked in bold. DN represents Dao Nong, NB = Na Bang, JHK = Ja Htu Kawng, MSY = Mung Seng Yang, SSL = Simsa Lawk

*Anopheles* mosquito density varied considerably among study sites (Table 2). Ja Htu Kawng had the highest *Anopheles* abundance with an average of 3.01 females per trap night (f/t/n), which was significantly higher than any other site; followed by Mung Seng Yang (0.76 f/t/n) and Na Bang (0.42 f/t/n). The high elevation site of Dao Nong (0.13 f/t/n) and the periurban site of Simsa Lawk (0.05 f/t/n) and had the lowest mosquito density.

**Table 2:** Population density (*Anopheles* females/trap/night) and species diversity of *Anopheles* mosquitoes at each study site

Village	Density (f/t/n) (95 % CI)	Species richness	Diversity index		Dominance index	Evenness
			Simpson	Shannon		
Ja Htu Kawng	3.01 [2.41, 3.60] a	18	0.18	0.50	0.91	0.17
Mung Seng Yang	0.76 [0.16, 1.35] b	20	0.44	1.16	0.74	0.39
Na Bang	0.42 [−0.43, 1.26] b	15	0.67	1.66	0.54	0.61
Dao Nong	0.13 [−0.74, 1.00] b	9	0.46	1.01	0.71	0.46
Simsa Lawk	0.05 [−0.55, 0.64] b	9	0.48	1.01	0.69	0.46

Different (same) letter indicating (no) significant difference at *p* = 0.05 level, Tukey HSD test of $\sqrt[4]{X}$ transformed data

*Anopheles* mosquito species composition varied considerably among study sites (Tables 1 and 2). Although *An. minimus* s.l. was dominant in four villages where elevation was low (54–91 %), *An. maculatus* s.l. was dominant in the high elevation site of Dao Nong village accounting for 71.3 % of all *Anopheles* collected (Tables 1 and 2). Other species that accounted for over 10 % in a given study site included *An. minimus* s.l. (14.7 %) in Dao Nong, *An. maculatus* s.l. (13.8 %) in Na Bang, and *An. culicifacies* (15.8 %) in Simsa Lawk (Table 1).

Na Bang had the highest species diversity, with a Simpson diversity index of 0.67, Shannon-Weiner index of 1.66 and 15 *Anopheles* species. It also had the highest evenness index (Table 2). Although 18 *Anopheles* species were detected, Ja Htu Kawng had the lowest species diversity (Simpson 0.18 and Shannon-Weiner 0.50) because *An. minimus* was so dominant (90.7 %) that other species contributed very little to the diversity, leading to the lowest evenness (Table 2). Despite very similar species diversity in the other three villages (diversity index 0.44–0.48), all 20 *Anopheles* species were captured in Mung Seng Yang, whereas only nine species were collected in both Dao Nong and Simsa Lawk villages (Table 2). Interestingly, similarity analysis showed that species composition in Dao Nong was significantly dissimilar to the rest of the villages (Morista-Horn indices < 0.43) while the other four villages had very similar species compositions (Morista-Horn indices range from 0.86 to 0.98) (Table 3).

**Table 3:** Similarity in species composition between different villages

	Dao Nong	Na Bang	Ja Htu Kawng	Mung Seng Yang	Simsa Lawk
Dao Nong	1				
Na Bang	0.43	1			
Ja Htu Kawng	0.21	0.86	1		
Mung Seng Yang	0.26	0.94	0.98	1	
Simsa Lawk	0.34	0.95	0.95	0.98	1

**Fig. 2:**
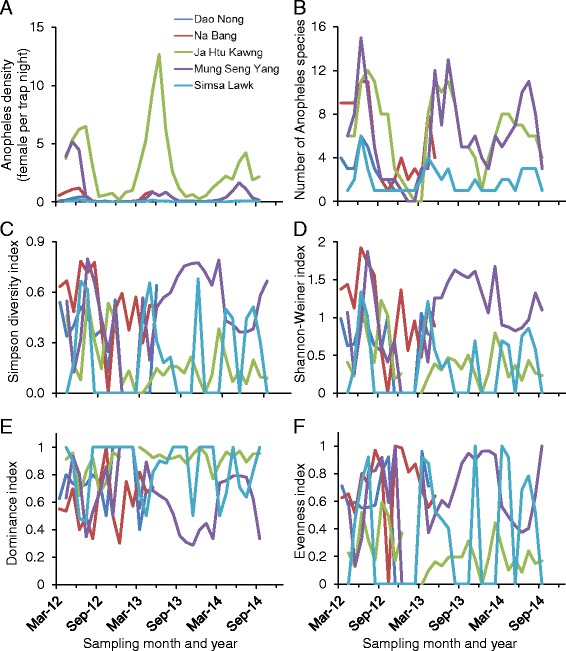
Population dynamics of *Anopheles* mosquitoes **a** population density (females/trap/night) pooled of all *Anopheles* species, **b** species richness, **c** Simpson species diversity, **d** Shannon-Weiner diversity index, **e** dominance index, and **f** evenness index in different stud sites

*Anopheles* population densities showed clear seasonality in all villages, with peak months from April to August each year (Fig. 2a). *Anopheles* population dynamics differed significantly amongst the study sites (repeated measure ANOVA *F*_4,114_ = 33.86, *P* < 0.0001). Ja Htu Kawng had significantly higher densities of *Anopheles* mosquitoes than the other villages, and differences in average monthly population densities among other villages was not statistically significant (Tukey-Kramer HSD, q* = 2.77, *P* < 0.05). Ja Htu Kawng had the highest density of any site in every month surveyed, and the periurban site of Simsa Lawk had more than 60-fold lower *Anopheles* density than Ja Htu Kawng.

Species richness, measured by the number of *Anopheles* species trapped, also showed strong seasonality in three study sites, Na Bang, Ja Htu Kawng, and Mung Seng Yang, but varied little in the other two sites (Fig. 2b). The periurban site, Simsa Lawk, and the high elevation site, Dao Nong, had the lowest number of *Anopheles* species, varying little over time; the other three sites had similar number of species all over the study period (Fig. 2b).

Interestingly, different from population density and species richness, species diversity fluctuated over time but did not show any seasonality in all study sites (Fig. 2c and d). Mung Seng Yang was the only village where species diversity was relatively stable and high in 2013 and 2014 (Fig. 2c and d). Similarly, dominance index and evenness indices varied substantially from time to time (Fig 2e and f). In Ja Htu Kawng and Simsa Lawk, dominance index was sometimes very high, i.e., with one species being dominant (Fig. 2e). In Ja Htu Kawng, dominance index was high most of the time, while evenness index was low in 2013 and 2014 (Fig. 2f).

## Discussion

Aside from Africa, Southeast Asia is the key focusing area of malaria transmission because of its intense *P. vivax* transmission and mixed infections. In addition, mosquito vectors show greater species diversity in Southeast Asia than in Africa. For example, in Africa, three *Anopheles* species, *An. gambiae* s.s., *An. funestus* s.l., and *An. arabiensis* are responsible for almost all malaria transmissions [27–29]. Conversely, there are more than 20 major malaria vector species in Southeast Asia, which makes vector control extremely difficult because of the complex biology and ecology of the different vectors [29–38]. Among the 20 identified *Anopheles* species in this study, l4 of them have been implicated in malaria transmission in East and Southeast Asia [29–38]. Furthermore, the top four most abundant *Anopheles* mosquitoes found in this study are all malaria vectors, including one of the key malaria vectors in Southeast Asia, *An. minimus* s.l., which was also the most abundant *Anopheles* species in the study area. This species is not exclusively anthropophilic or zoophilic, not exclusively endophagic or exophagic, and not exclusively exophilic or endophilic, therefore, it is extremely difficult to control it with currently available tools [17, 39, 40]. While other countries have implemented intensive vector-borne disease control efforts and have seen a decline in the prevalence of malaria, the malaria situation in Myanmar is largely unknown [6–8]. It is believed that Myanmar is and will remain the key malaria parasite reservoir in Southeast Asia, significantly offsetting the enormous regional efforts for malaria elimination [7–9]. Studies found that the majority of the *P. falciparum* malaria cases in China were imported from Myanmar [14, 41, 42]. To effectively control malaria in Myanmar and to eliminate malaria in China, it is essential to understand the community structure and species composition, the temporal dynamics, and the spatial distribution of mosquito vectors in this area.

The seasonal fluctuations in vector density and species richness is likely due to the seasonal rainfalls and temperature changes [43, 44]. Although there is no clear dry season in the study area, summer time (May to August) is usually the rainy season, when temperatures can reach 25–30 °C, which is perfect for malaria transmission [44, 45]. Winter is relatively dry and temperatures drop to an average of around 10–15 °C, which is unfavorable to the development of both immature and adult mosquitoes. This climatic-vector interaction is common in *Culex*, *Aedes*, and *Anopheles* mosquitoes, for example, vector population dynamics are strongly associated with climatic conditions, winter diapause or refuge, and water availability affecting mosquito development [46–49]. This seasonality in vector abundance leads to seasonality of vector-borne infectious diseases [41, 42, 45, 49–51].

Differences in vector abundance, community structure, and *Anopheles* species composition at different sites may be explained by the contextual determinants, mainly environmental factors. There are many water ponds in Na Bang, Ja Htu Kawng, and Mung Seng Yang villages. Residents in these villages raise a lot of livestock such as pigs and bovines (mainly domestic cattle and water buffalo), providing a perfect environment for *Anopheles* mosquitoes, especially the zoo-anthropophilic species *An. minimus*. Larvae of *An. sinensis* (in Na Bang) and *An. minimus* s.l. (in Ja Htu Kawng) were frequently found in these villages (GZ unpublished data). On the other hand, in both Dao Nong and Simsa Lawk villages, abundance of *Anopheles* was very low. Both villages have heavy forest coverage, although with totally different settings – rural in Dao Nong vs. urban in Simsa Lawk, and both have fewer livestock than the other three villages. *An. minimus* s.l. was dominant in Ja Htu Kawng, but both Na Bang and Mung Seng Yang had a diverse *Anopheles* composition. These differences in *Anopheles* abundance and species composition suggest that micro-environmental factors may play an important role in influencing the occurrence and abundance of these species.

It is worth mentioning that the result from this study is different from the result of the study conducted in northern Thailand [37], which found that *An. minimus* s.l. occurs in less disturbed forested area and that deforestation has negative effects on *An. minimus* s.l. abundance [38]. In our study, *An. minimus* s.l. was most abundant in deforested areas such as Ja Htu Kawng and Mung Seng Yang villages and it was less abundant in the forested areas such as Na Bang and Dao Nong. In the Thailand study, breeding habitats were mainly found along streams outside the villages, whereas in this study, habitats were mainly standing water ponds within the village (GZ personal observations), we suspect that the species found in the two studies are different. Unfortunately, the Thailand study did not mention details about the *An. minimus* s.l. species. Most of the *Anopheles* mosquitoes caught (97 %) were known malaria vectors, including the key malaria vectors in Southeast Asia *An. minimus* s.l.. *Anopheles sinensis* is considered the most common malaria vector in China [32], but it is not common in the study area, perhaps because *An. sinensis* prefers rice paddies and does not like the forest environment. In addition to inter-villages variations in *Anopheles* densities and species diversity, there is the potential of intra-village heterogeneity, such as house-to-house variations in *Anopheles* densities and possible spatial clustering, which is open for further investigation.

## Conclusion

In conclusion, this study provides fundamental information on mosquito abundance and population dynamics in the China-Myanmar border area. The results indicate that both vector abundance and species diversity vary greatly from village to village and from season to season. The knowledge gained from this study will be useful for designing targeted intervention strategies for malaria control in Myanmar and malaria elimination in China.

## Additional file

Additional file 1:
**GPS readings of all surveyed houses.** (XLSX 18 kb)

## References

[1] World Health Organization (1995). Vector control for malaria and other mosquito-borne disease.

[2] Morrison AC, Zielinski-Gutierrez E, Scott TW, Rosenberg R (2008). Defining challenges and proposing solutions for control of the virus vector *Aedes aegypti*. PLoS Med.

[3] mal ERACGoVC (2011). A research agenda for malaria eradication: vector control. PLoS Med.

[4] van den Hurk AF, Ritchie SA, Mackenzie JS (2009). Ecology and geographical expansion of Japanese encephalitis virus. Annu Rev Entomol.

[5] Tomori O (2002). Yellow fever in Africa: public health impact and prospects for control in the 21st century. Biomedica.

[6] World Health Organization (2012). World malaria report 2012.

[7] Cui L, Yan G, Sattabongkot J, Cao Y, Chen B, Chen X (2012). Malaria in the Greater Mekong Subregion: heterogeneity and complexity. Acta Trop.

[8] Delacollette C, D’Souza C, Christophel E, Thimasarn K, Abdur R, Bell D (2009). Malaria trends and challenges in the Greater Mekong Subregion. Southeast Asian J Trop Med Public Health.

[9] Gething PW, Elyazar IR, Moyes CL, Smith DL, Battle KE, Guerra CA (2012). A long neglected world malaria map: *Plasmodium vivax* endemicity in 2010. PLoS Negl Trop Dis.

[10] Battle KE, Gething PW, Elyazar IR, Moyes CL, Sinka ME, Howes RE (2012). The global public health significance of *Plasmodium vivax*. Adv Parasitol.

[11] World Health Organization (2008). World malaria report 2008.

[12] World Health Organization (2010). Malaria in the Great Mekong Subregion: Regional and Country Profile. WHO Southeast Asia and Western Pacific Region, India.

[13] World Health Organization (2010). World malaria report 2010.

[14] Xia ZG, Yang MN, Zhou SS (2012). Malaria situation in the People’s Republic of China in 2011. Chin J Parasitol Parasitic Dis.

[15] Lin H, Lu L, Tian L, Zhou S, Wu H, Bi Y (2009). Spatial and temporal distribution of *falciparum* malaria in China. Malar J.

[16] Yan J, Li N, Wei X, Li P, Zhao Z, Wang L (2013). Performance of two rapid diagnostic tests for malaria diagnosis at the China-Myanmar border area. Malar J.

[17] Yu G, Yan G, Zhang N, Zhong D, Wang Y, He Z (2013). The *Anopheles* community and the role of *Anopheles minimus* on malaria transmission on the China-Myanmar border. Parasit Vectors.

[18] Zhou X, Li SG, Chen SB, Wang JZ, Xu B, Zhou HJ (2013). Co-infections with *Babesia microti* and *Plasmodium* parasites along the China-Myanmar border. Infect Dis Poverty.

[19] Moore SJ, Min X, Hill N, Jones C, Zaixing Z, Cameron MM (2008). Border malaria in China: knowledge and use of personal protection by minority populations and implications for malaria control: a questionnaire-based survey. BMC Public Health.

[20] Dong SH (2010). The mosquito fauna of Yunnan.

[21] Dong SH, Zhou HN, Gong ZD (2010). The mosquito fauna of Yunnan.

[22] Alatalo R, Alatalo R (1977). Components of diversity: multivariate analysis with interaction. Ecology.

[23] Norris JL, Pollock KH (1998). Non-parametric MLE for Poisson species abundance models allowing for heterogeneity between species. Environ Ecol Stat.

[24] Kweka EJ, Zhou G, Munga S, Lee MC, Atieli HE, Nyindo M (2012). *Anopheline* larval habitats seasonality and species distribution: a prerequisite for effective targeted larval habitats control programmes. PLoS One.

[25] Southwood TRE, Henderson PA (2000). Ecological methods. Blackwell science.

[26] Legendre P, Legendre L (1998). Numerical ecology.

[27] Zhou G, Afrane YA, Vardo-Zalik AM, Atieli H, Zhong D, Wamae P (2011). Changing patterns of malaria epidemiology between 2002 and 2010 in western Kenya: the fall and rise of malaria. PLoS One.

[28] Sinka ME, Bangs MJ, Manguin S, Rubio-Palis Y, Chareonviriyaphap T, Coetzee M (2012). A global map of dominant malaria vectors. Parasit Vectors.

[29] Sinka ME, Bangs MJ, Manguin S, Chareonviriyaphap T, Patil AP, Temperley WH (2011). The dominant *Anopheles* vectors of human malaria in the Asia-Pacific region: occurrence data, distribution maps and bionomic precis. Parasit Vectors.

[30] Elyazar IR, Sinka ME, Gething PW, Tarmidzi SN, Surya A, Kusriastuti R (2013). The distribution and bionomics of *Anopheles* malaria vector mosquitoes in Indonesia. Adv Parasitol.

[31] Alam MS, Khan MG, Chaudhury N, Deloer S, Nazib F, Bangali AM (2010). Prevalence of Anopheline species and their *Plasmodium* infection status in epidemic-prone border areas of Bangladesh. Malar J.

[32] Zhu G, Xia H, Zhou H, Li J, Lu F, Cao J, Gao Q (2013). Susceptibility of *Anopheles sinensis* to *Plasmodium vivax* in malarial outbreak areas of central China. Parasit Vectors.

[33] Zollner GE, Ponsa N, Garman GW, Poudel S, Bell JA, Sattabongkot J (2006). Population dynamics of sporogony for *Plasmodium vivax* parasites from western Thailand developing within three species of colonized *Anopheles* mosquitoes. Malar J.

[34] Singh N, Chand SK, Bharti PK, Singh MP, Chand G, Mishra AK (2013). Dynamics of forest malaria transmission in Balaghat district, Madhya Pradesh, India. PLoS One.

[35] Bashar K, Tuno N, Ahmed TU, Howlader AJ (2013). False positivity of circumsporozoite protein (CSP)-ELISA in zoophilic anophelines in Bangladesh. Acta Trop.

[36] Rattanarithikul R, Konishi E, Linthicum KJ (1996). Detection of *Plasmodium vivax* and *Plasmodium falciparum* circumsporozoite antigen in anopheline mosquitoes collected in southern Thailand. Am J Trop Med Hyg.

[37] Overgaard HJ, Tsuda Y, Suwonkerd W, Takagi M (2002). Characteristics of *Anopheles minimus* (Diptera: Culicidae) larval habitats in Northern Thailand. Environ Entomol.

[38] Garros C, Koekemoer LL, Coetzee M, Coosemans M, Manguin S (2004). A single multiplex assay to identify major malaria vectors within the African *Anopheles funestus* and the Oriental *An. minimus* groups. Am J Trop Med Hyg.

[39] Chareonviriyaphap T, Bangs MJ, Suwonkerd W, Kongmee M, Corbel V, Ngoen-Klan R (2013). Review of insecticide resistance and behavioral avoidance of vectors of human diseases in Thailand. Parasit Vectors.

[40] Hii J, Rueda LM (2013). Malaria vectors in the Greater Mekong Subregion: overview of malaria vectors and remaining challenges. Southeast Asian J Trop Med Public Health.

[41] Li N, Parker DM, Yang Z, Fan Q, Zhou G, Ai G (2013). Risk factors associated with slide positivity among febrile patients in a conflict zone of north-eastern Myanmar along the China-Myanmar border. Malar J.

[42] Zhou G, Sun L, Xia R, Duan Y, Xu J, Yang H (2014). Clinical malaria along the China-Myanmar border, Yunnan Province, China, January 2011-August 2012. Emerg Infect Dis.

[43] Bouma MJ (2003). Methodological problems and amendments to demonstrate effects of temperature on the epidemiology of malaria. A new perspective on the highland epidemics in Madagascar, 1972–89. Trans R Soc Trop Med Hyg.

[44] Kristan M, Abeku TA, Beard J, Okia M, Rapuoda B, Sang J (2008). Variations in entomological indices in relation to weather patterns and malaria incidence in East African highlands: implications for epidemic prevention and control. Malar J.

[45] Paaijmans KP, Blanford S, Bell AS, Blanford JI, Read AF, Thomas MB (2010). Influence of climate on malaria transmission depends on daily temperature variation. PNAS.

[46] Sithiprasasna R, Linthicum KJ, Liu GJ, Jones JW, Singhasivanon P (2003). Some entomological observations on temporal and spatial distribution of malaria vectors in three villages in northwestern Thailand using a geographic information system. Southeast Asian J Trop Med Public Health.

[47] Ebrahimi B, Shakibi S, Foster WA (2014). Delayed egg hatching of *Anopheles gambiae* (Diptera: Culicidae) pending water agitation. J Med Entomol.

[48] Tsunoda T, Cuong TC, Dong TD, Yen NT, Le NH, Phong TV (2014). Winter refuge for *Aedes aegypti* and *Ae. albopictus* mosquitoes in Hanoi during Winter. PLoS One.

[49] Tian HY, Bi P, Cazelles B, Zhou S, Huang SQ, Yang J (2015). How environmental conditions impact mosquito ecology and *Japanese encephalitis*: an eco-epidemiological approach. Environ Int.

[50] Midekisa A, Beyene B, Mihretie A, Bayabil E, Wimberly MC (2015). Seasonal associations of climatic drivers and malaria in the highlands of Ethiopia. Parasit Vectors.

[51] Srimath-Tirumula-Peddinti RC, Neelapu NR, Sidagam N (2015). Association of climatic variability, vector population and malarial disease in District of Visakhapatnam, India: a modeling and prediction analysis. PLoS One.

